# Chemical synthesis and mechanism of a natural product from endolichenic fungus with a broad-spectrum anti microorganism activity

**DOI:** 10.3389/fmicb.2023.1168386

**Published:** 2023-05-05

**Authors:** Xuan Zhou, Ming-Yi Wang, Qian-Ping Cao, Ze Yang, Qing-Feng Meng, Shao-Bin Fu

**Affiliations:** ^1^School of Pharmacy, Zunyi Medical University, Zunyi, China; ^2^School of Public Health, Zunyi Medical University, Zunyi, China; ^3^Centre of Excellence in Fungal Research, Mae Fah Luang University, Chiang Rai, Thailand

**Keywords:** natural product, endolichenic fungus, anti-microorganism activity, mechanism, chemical synthesis

## Abstract

**Background:**

The antibiotic resistance in various bacteria is consistently increasing and is posing a serious threat to human health, prompting the need for the discovery of novel structurally featured natural products with promising biological activities in drug research and development. Endolichenic microbes have been proven to be a fertile source to produce various chemical components, and therefore these microbes have been on a prime focus for exploring natural products. In this study, to explore potential biological resources and antibacterial natural products, the secondary metabolites of an endolichenic fungus have been investigated.

**Methods:**

The antimicrobial products were isolated from the endolichenic fungus using various chromatographic methods, and the antibacterial and antifungal activities of the compounds were evaluated by the broth microdilution method under *in vitro* conditions. The antimicrobial mechanism has been discussed with measuring the dissolution of nucleic acid and protein, as well as the activity of alkaline phosphatase (AKP) in preliminary manner. Chemical synthesis of the active product compound 5 was also performed, starting from commercially available 2,6-dihydroxybenzaldehyde through a sequence of transformations that included methylation, the addition of propylmagnesium bromide on formyl group, the oxidation of secondary alcohol, and the deprotection of methyl ether motif.

**Results:**

Among the 19 secondary metabolites of the endolichenic fungus, *Daldinia childiae* (compound 5) showed attractive antimicrobial activities on 10 of the 15 tested pathogenic strains, including Gram-positive bacteria, Gram-negative bacteria, and fungus. The Minimum Inhibitory Concentration (MIC) of compound 5 for *Candida albicans* 10213, *Micrococcus luteus* 261, *Proteus vulgaris* Z12, *Shigella sonnet*, and *Staphylococcus aureus* 6538 was identified as 16 μg/ml, whereas the Minimum Bactericidal Concentration (MBC) of other strains was identified as 64 μg/ml. Compound 5 could dramatically inhibit the growth of *S. aureus* 6538, *P. vulgaris* Z12, and *C. albicans* 10213 at the MBC, likely affecting the permeability of the cell wall and cell membrane. These results enriched the library of active strains and metabolites resources of endolichenic microorganisms. The chemical synthesis of the active compound was also performed in four steps, providing an alternative pathway to explore antimicrobial agents.

## 1. Introduction

The antibiotic resistance in various bacteria is consistently increasing and is posing a serious threat to human health. Due to the overuse or abuse of antibiotics, pathogenic bacteria have developed resistance to a variety of antibacterial drugs, posing a serious threat to global public health and economic stability. Drug resistance has become one of the most pressing global public health issues, making it imperative to find new antibacterial agents (Seukepa et al., 2022; Shinu et al., 2022). To provide chemical entities for the current rigorous drug research and development, new natural products with novel structures and significant activity are urgently needed. Antibiotics such as penicillin, streptomycin, erythromycin, and many others were discovered from microorganisms. According to the literature, 12 out of the 22 antibacterial drugs marketed after 2,000 originated from microbial secondary metabolites. In the global drug market, approximately 70% of antibiotics are derived from microorganisms (Rao et al., 2020). Microbial natural products have always been the main source of biological drug innovation, and are important resources for developing clinical antibacterial, anti-tumor, immunosuppressant, and other drugs which are also widely used in agriculture, food, manufacturing industry, and other fields (Anderson et al., 2022).

However, the problem of repeated screening of microbial resources has become increasingly prominent as the intense research on secondary metabolites from microorganisms (Maliehe et al., 2022). Obviously, the probability of the discovery of novel structural active substances from microbes in conventional environments has gradually declined. It is crucial to actively search for new microbial resources to advance the research and development of natural product drugs (Nursyam, 2017; Shevkar et al., 2022).

Lichens are considered as stable complex organisms formed by the symbiosis of fungi and algae. Through the long process of biological evolution, mycobionts, photobionts, and endolichenic fungi have developed a special symbiotic relationship. Due to their distinctive ecological environments, it is widely believed that lichens and endophytes contain a diverse array of unique active metabolites (Zhao et al., 2022). Progressively more novel and bioactive compounds have been reported from endolichenic fungi in recent years (Lin et al., 2021; Luo et al., 2022). Guizhou Province of Southwestern China is a typical mountainous region with a high level of species diversity owing to its unique Karst terrain and humid climate.

The endolichenic fungus, *D. childiae*, from *Punticelia* sp. isolated by our research team showed strong- and broad-spectrum antibacterial activities on *Staphylococcus aureus* 25923 and 6538, *Staphylococcus epidermidis* Z12, *Bacillus subtilis* 163, and so on (Zhou et al., 2021a). In this fungus, a new compound and several first-isolated natural products were discovered (Zhou et al., 2020, 2021b). Moreover, it was exciting to observe that a product 1-(2,6-dihydroxyphenyl) butane-1-one (5) exhibited inhibitory activity on 10 pathogenic bacteria among 15 strains provided in clinical and food fields. In this study, the antibacterial, antifungi activities, and anti-microorganism mechanisms were investigated. Furthermore, in this study, the chemical synthesis was explored. The results from this study indicate that endolichenic fungi are valuable and potential resources for exploiting new antibacterial substances as the candidates in the clinical application and in preserving the food safety.

## 2. Materials and methods

### 2.1. General experimental procedures

The Nuclear Magnetic Resonance (NMR) data were detected by the DD2400-MR Nuclear Magnetic Resonance Instrument (Agilent, Santa Clara, CA, USA). Preparative High-Performance Liquid Chromatography (Preparative HPLC) was performed on the LC3000N Liquid Chromatography System, equipped with a C18 (5 μm 10 × 250 mm, HPLC semipreparative column) (Column Company, Seattle, WA, USA) and a UV3000 Ultraviolet–Visible Light Detector. The absorbance of the substances was detected by a Microplate Reader (MK3 Thermo Company, Pleasanton, CA, USA). Sterile conditions were provided by an Aseptic ultra-clean bench (Suzhou Purification Equipment Co., Ltd., Suzhou, China). The thermostatic shaker is manufactured by Shanghai Tiancheng Technology Co., Ltd. (Shanghai, China). Concentrations in the suspension of the strain were identified by Bacterial Turbidity Meter (WGZ-2XJ, Shanghai, China). Mueller–Hinton (MH) broth was purchased from Beijing Solabao Technology Co., Ltd., (Beijing, China) while yeast extract and peptone were purchased from Guangzhou Lu Cheng Biotechnology Co., Ltd. (Guangzhou, China). Other analytical-grade solvents were purchased from Tianjin Fuyu Fine Chemical Co., Ltd., Tianjin, China.

### 2.2. Fungal materials

The fungal strain *D. childiae* isolated from the *Punticelia* was identified by the Internal Transcribed Spacer (ITS) sequence (GenBank No. MT139887) and deposited in China General Microbiological Culture Collection Center (CGMCC No. 17469). A total of 15 pathogenic bacterial and fungal strains including Gram-positive bacteria, Gram-negative bacteria, and fungi were provided. *Listeria monocytogenes* CMCC 54008, *Salmonella typhimurium* CMCC 50071, *Vibrio parahaemolyticus* ATCC 17802, *Pseudomonas aeruginosa*, *Enterobacter aerogenes*, *Vibrio vulnificus*, *Proteus mirabilis*, *Shigella sonnei*, *Shigella sonnet* were provided by School of Public Health, Zunyi Medical University, Guizhou, China. *Brucella* sp. B103, *Proteus vulgaris* Z12, *B. subtilis* 163 and *Micrococcus luteus* 261 were provided by Affiliated Hospital, Zunyi Medical University, Guizhou, China. *S. aureus* 6538 and *Candida albicans* 10213 were provided by the School of Pharmacy, Zunyi Medical University, Guizhou, China.

### 2.3. The isolation of compound 5 and inhibitory activity on pathogenic strains

Compound 5 was isolated from the rice solid fermentation extract obtained from ethyl acetate through silica gel column chromatography (Zhou et al., 2021b). The antibacterial activity of compound 5 was evaluated by the filter paper method.

#### 2.3.1. Preparation of culture media

Mueller–Hinton liquid media (9.125 g MHA powder in 250 ml distilled water) and Luria-Bertani (LB) liquid media (yeast extract, NaCl, and peptone in the ratio of 1 g: 1 g:2 g in 200 ml distilled water) were prepared and the solid media were formed with addition with 20-g ager into 1 L liquid media.

#### 2.3.2. Activation of test strains

The 15 tested strains were inoculated on the sterile LB solid media and incubated at 37°C for 12 h to obtain a single colony. Then the single colony was picked to add the LB liquid media and incubated at 37°C at 150 rpm for another 12 h.

#### 2.3.3. Screening of the active compounds on tested strains

Nineteen isolated compounds were screened for their antibacterial and antifungal activities using the Kirby–Bauer method. Fifteen test strains of bacteria and fungi were inoculated on MHA media, and drug-sensitivity papers were placed on the plates. Then, 1.5 mg of the compound was dissolved in 500 μl of methanol and 5 μl of the solution of each compound was placed on the filter paper, with methanol as the blank control. The treated strains were incubated at 37°C with three replicates. After 18 h, the inhibition zones were measured to evaluate the antibacterial and anti-fungal effects of the isolated secondary metabolites.

### 2.4. MIC and MBC of compound 5 by microdilution method

#### 2.4.1. Preparation of solutions of compound 1 and pathogenic strains

An amount of 2 mg of compound 5 was added to 1 ml of methanol in a 1.5 ml Eppendorf (EP) tube and mixed thoroughly. Then, 224 μl of the resulting solution was added to 126 μl of broth and further diluted to a concentration of 1,280 μg/ml. The concentration of the final solution was achieved by continuous dilution to 1.25 μg/ml.

The bacterial solution was diluted to 0.5 Maxwell’s turbidity with the turbidimetric tube as a reference, which is about 1 × 10^8^ CFU/ml by the broth. Subsequently, 950 μl of MH culture media and 50 μl of bacterial solution with 0.5 Maxwell’s turbidity were mixed in a new EP pipette to make the bacterial suspension with an approximate concentration of 1 × 10^7^ CFU/ml.

#### 2.4.2. Determination of MIC

The MICs of the compounds were determined by the broth microdilution method (Clarke et al., 2020). Initially, 180 μl of broth culture media were added to the holes 1–10 in each row, while 190 μl of broth culture media were added to the holes 11–12 as the control in a sterile 96-well plate. Subsequently, 10 μl of bacterial solution was added to the holes 1–11 and 10 μl of the prepared drug solution was added to the holes 1–10 of each row. Additionally, 10 μl of the minimum concentration solution was added to the hole 12. The tests were repeated 3 times in parallel. All 96-well plates were incubated for 24–36 h at 37°C. The MIC of the drug was determined as the one with a clear and transparent solution and in the other holes, it was observed with a black background, while that of the negative control was observed as turbid liquid.

#### 2.4.3. Determination of MBC

Based on the MIC, 10 μl of culture solution in which the test bacteria did not grow from a 96-well plate were incubated on the MHA media without drug solution at 37°C for 24–36 h after coating. According to Liu et al. (2015), the concentration of the compound without the growth of test bacteria is considered as the MBC value.

### 2.5. Determination of growth curve of test bacteria

#### 2.5.1. Activation of test bacteria

First, three strains including Gram-positive bacteria, *S. aureus* 6538; Gram-negative bacteria, *P. vulgaris* Z12; and fungus, *C. albicans* 10213, were activated from the stored plate at –20°C on the LB solid media for 12 h at 37°C. The single colony was picked out and put into the LB liquid media for another 12 h at 37°C in a shaker at 150 rpm.

#### 2.5.2. Determination of growth curve

According to the method by Wu et al. (2017), the activated test bacterial solution was diluted with MH broth to a concentration of 10^7^ CFU/ml, and then 200 μl of the diluted test bacterial solution was taken into a flask containing 20 ml sterile nutrient broth. To achieve a final concentration of 1 × MBC, compound 5 was dissolved in methanol and added to the solution. The control group was set up with MH broth that did not contain the compound but contained the solvent and the bacterial solution being tested. All the bacterial solutions were incubated at 37°C and 120 rpm, and the growth of the test bacteria was monitored every 2 h under OD_600_ on the enzyme-labeled instrument.

### 2.6. The content of nucleic acid

#### 2.6.1. Preparation of PBS buffer solution

A total amount of 17.4170 g of K_2_HPO_4_ and 13.609 g of KH_2_PO_4_ were added into a 100 ml volumetric flask to make the solutions. Then a 50.3 ml of KH_2_PO_4_ and 49.7 ml of K_2_HPO_4_ were mixed in a new 250 ml triangular flask. The PBS buffer solution with pH 6.8 was prepared after sterilization of the mixed liquids at 121°C for 15 min.

#### 2.6.2. Determination of the content of nucleic acid

The content of nucleic acid was determined by the improved method used by Zhang et al. (2020). The test bacterial suspension with a concentration of 10^7^ CFU/ml was inoculated into 100 ml MH broth into the logarithmic phase. Then 10 ml of bacterial suspension was taken into a 50 ml centrifuge tube and centrifuged at 2,700 *g* for 30 min and the bacterial suspension was washed 3 times with PBS buffer solution. To obtain a final concentration of 1 × MBC, 100 μl of compound 5 was dissolved in methanol and was added to the solution, followed by the addition of MH broth to bring the final volume to 10 ml. After 3 h at 37°C, the bacterial suspension was centrifuged at 2,700 *g* for 15 min. To measure the concentration of the extracted nucleic acid, 200 μl of the supernatant was transferred to a 96-well plate, and the absorbance at OD_260_ was recorded using a microplate reader. The control group was set up with only methanol but without the compound. The phosphate-buffered saline (PBS) buffer solution and MH broth were used for blank correction, and the determination was performed in triplicate.

### 2.7. Determination of the content of protein

The supernatant was obtained according to the method of determination of nucleic acid, and the content of protein was determined by the method by Chen et al. (2020). For the bicinchoninic acid (BCA) determination, the working solution and diluted BSA standard solution were prepared according to the instructions suggested by the assay kit used (Solarbio, China). The standard curve was drawn based on the value of OD_562_ with the enzyme-labeled instrument. All the measurements were repeated 3 times in parallel and the protein concentration was calculated by the standard curve. The control group was treated in the same manner as the compound group except for the addition of the compound. Both the compound group and the control group were subjected to the same experimental conditions to ensure any observed effects, due to the presence or absence of the compound, were solely being tested.

### 2.8. Determination of content of AKP

The content of AKP was determined by the modified method (He et al., 2018). A compound background group was set to deduct the interference of the compound itself in the experiment. The supernatant was obtained by the same method followed in the determination of nucleic acid and measured at optical density (OD_520_) on the microplate reader according to the instructions of the kit. The measurements were repeated 3 times in parallel and then the AKP in the liquid was calculated according to the following formula:


$$AKPinculturemedia=\frac{\mathrm{OD}\,\mathrm{test}-\mathrm{OD}\,\mathrm{blank}\,\mathrm{control}}{\mathrm{OD}\,\mathrm{standard}-\mathrm{OD}\,\mathrm{blank}\,\mathrm{control}}\times$$

Standardconcentrationofphenol(0.02mg/ml)×100ml×

D⁢i⁢l⁢u⁢t⁢i⁢o⁢n⁢r⁢a⁢t⁢i⁢o

Definition: A volume of 100 ml liquid reacts with the matrix for 15 min to produce 1 mg of phenol at 37°C, which is defined as 1 Guinness unit.

### 2.9. Statistical analysis

The Microsoft Excel 2019 and SPSS 17 software were used for the statistical analysis of the data. The results were expressed in [± standard deviation (SD)], and *p* < 0.05 was statistically significant.

### 2.10. Synthesis of 2-hydroxy-6-methoxybenzaldehyde (compound 2)

To a stirred mixture of 2,6-dihydroxybenzaldehyde (690.6 mg, 5 mmol, 1.0 equiv.), and K_2_CO_3_ (2.764 g, 20 mmol, 4.0 equiv.) in tetrahydrofuran (THF) (30 ml), CH_3_I (2.280 g, 1 ml, 15 mmol, 2 equiv.) was added slowly dropwise at room temperature. When the 2,6-dihydroxybenzaldehyde was consumed completely, which was detected by thin-layer chromatography (TLC) (petroleum ether: ethyl acetate in the ratio of 15:1, v/v), the resulting mixture was quenched with saturated aqueous NH_4_Cl (5 ml). Reaction conditions, including reaction temperature, reaction time, and solvents, are presented in Table 1. Then, the mixture was filtered, and extracted with ethyl acetate (5 ml × 3). The combined organic phases were dried over anhydrous Na_2_SO_4_, filtered, and concentrated under reduced pressure. The residue was purified by column chromatography on silica gel with petroleum ether: ethyl acetate in the ratio of 30:1 (v/v) to afford 538.6 mg white solid of compound 2 in 78% yield. ^1^H NMR (400 MHz, CDCl_3_) (Supplementary Figure 1): δ 11.85 (s, 1H), 10.31 (s, 1H), 7.38 (t, *J* = 8.4 Hz, 1H), 6.49 (d, 1H), and 6.36 (d, *J* = 8.3, 0.9 Hz, 1H), 3.86 (s, 3H).

**TABLE 1:** The reaction conditions for the synthesis of 2-hydroxy-6-methoxybenzaldehyde.

Entry	Substrate	Temp (°C)	Time (h)	Solvent	Yield (%)
1	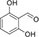	RT	3	THF	30
2	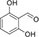	68	3	THF	20
3	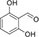	RT	6	THF	78
4	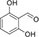	68	6	THF	60
5	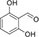	RT	6	DMF	25
6	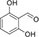	80	6	DMF	28
7	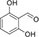	RT	6	DCM	10

### 2.11. Synthesis of 1 (2-hydroxy, 6-methoxyphenyl) butanol (compound 3)

Under the argon atmosphere, in an oven-dried 100 ml two-neck flask equipped with a magnetic stir bar, 2-hydroxy, 6-methoxybenzaldehyde (634 mg, 3.83 mmol) and anhydrous tetrahydrofuran (5 ml) were added. The reaction mixture was cooled to 0°C with ice–water bath. Then an 8 ml propylmagnesium bromide (8.0142 g, 7.66 mmol) was added into the reaction mixture dropwise and stirred at 0°C for further 6 h. The mixture was quenched with saturated NH_4_Cl solution. Reaction conditions, including reaction temperature, reaction time, and solvents, are presented in Table 2. Then, the resulting mixture was extracted with ethyl acetate (5 ml × 3). The combined organic phases were dried over anhydrous Na_2_SO_4_, filtered, and concentrated under reduced pressure. The residue was purified by column chromatography using petroleum ether: ethyl acetate in the ratio of 10:1 (v/v) as eluent to afford 443.8 mg light yellow oil in 70% yield. ^1^H NMR (400 MHz, CDCl_3_) (Supplementary Figure 2): δ 8.72 (s, 1H), 7.08 (t, *J* = 8.2 Hz, 1H), 6.49 (d, *J* = 8.2 Hz, 1H), 6.38 (d, *J* = 8.2 Hz, 1H), 5.33 (td, *J* = 5.8, 5.3, 2.8 Hz, 1H), 3.73 (s, 3H), 2.91 (s,1H), 2.33—2.31 (m, 2H), 1.69 (m, 2H), and 0.93 (t, *J* = 7.4 Hz, 3H). ^13^C NMR (100 MHz, CDCl_3_) (Supplementary Figure 3): δ 156.97, 156.63, 128.70, 116.07, 110.21, 102.13, 70.14, 55.59, 38.60, 18.87, 13.98.

**TABLE 2:** The conditions of synthesis of 1-(2-hydroxy-6-methoxyphenyl) butanol.

Entry	Substrate	Temp (°C)	Time (h)	Solvent	Yield (%)
1	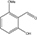	0	3	THF	30
2	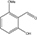	−20	3	THF	25
3	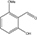	−78	3	THF	15
4	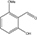	0	6	y THF	70
5	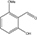	−20	6	THF	55
6	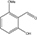	−78	6	THF	40

### 2.12. Synthesis of 1-(2-hydroxy-6-methoxyphenyl) butanone (compound 4)

In an oven-dried 100 ml round-bottomed flask equipped with a magnetic stir bar, 1-(2-hydroxy-6-methoxyphenyl)butanol (69 mg, 0.35 mmol) was dissolved in anhydrous dichloromethane (10.0 ml). Then Pyridine and CrO_3_ complex salt (PCC) (in hydrochloric acid solution) (75.5 mg, 0.35 mmol) was added, and the mixture was stirred at room temperature for 2 h. After the transformation was completed, the reaction mixture was filtered and concentrated under reduced pressure. Purification of the residue through column chromatography on silica gel using a gradient mixture of petroleum ether: ethyl acetate (in the ratio ranging from 80:1 to 50:1, v/v) and afforded 20.7 mg white solid 1-(2-hydroxy, 6-methoxyphenyl) butanone in 30% yield. ^1^H NMR (400 MHz, CDCl_3_) (Supplementary Figure 4): δ 13.30 (s, 1H), 7.33 (t, *J* = 8.3 Hz, 1H), 6.57 (d, *J* = 8.9 Hz, 1H), 6.39 (d, *J* = 8.3 Hz, 1H), 3.90 (s, 3H), 3.03 (t, *J* = 7.3 Hz, 2H), 1.71 (m, *J* = 7.4 Hz, 2H), and 0.99 (t, *J* = 7.4 Hz, 3H). Reaction conditions, including reaction temperature, reaction time, and solvents, are presented in Table 3.

**TABLE 3:** The conditions of synthesis of 1-(2-hydroxy-6-methoxyphenyl) butanone.

Entry	Substrate	Time (h)	Oxidant	Yield (%)
1	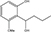	2	PCC	30
2	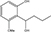	4	PCC	27
3	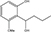	6	PCC	25
4	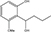	2	IBX	15
5	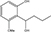	4	IBX	18
6	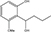	6	IBX	20
7	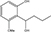	2	DMP	17

### 2.13. Synthesis of 1-(2,6-dihydroxyphenyl) butane-1-one (compound 5)

In an oven-dried 100 ml two-necked flask equipped with a magnetic stir bar, 1 (2-hydroxy6-methoxyphenyl)butanone (40 mg, 0.2 mmol, 1.0 equiv.) was dissolved in dry dichloromethane (2.0 ml) under an argon atmosphere. The solution was cooled to 0°C with an ice–water bath and BBr_3_ (50 μl, 0.6 mmol, 3 equiv.) was dropwise added into the solution. Then, the reaction mixture was stirred for 10 h at the same temperature. After that, the reaction solution was warmed to room temperature, diluted with dichloromethane (10 ml), and poured into ice water. The resulting mixture was extracted with dichloromethane (5 ml × 3). The combined organic phases were dried over anhydrous Na_2_SO_4_, filtered, and concentrated under reduced pressure. The residue was purified by column chromatography on silica gel using petroleum ether: ethyl acetate in the ratio of 10:1 (v/v) as eluent to afford 20.5 mg white solid of 1-(2,6-dihydroxyphenyl)butane-1-one in 51% yield. ^1^H NMR (400 MHz, CDCl_3_) (Supplementary Figure 5): δ 9.82 (s, 2H), 7.22 (t, *J* = 8.2 Hz, 1H), 6.40 (d, *J* = 8.1 Hz, 2H), 3.13 (t, *J* = 14.6 Hz, 2H), 1.75 (m, 2H), and 1.01 (t, *J* = 7.4 Hz, 3H). ^13^C NMR (100 MHz, CDCl_3_) (Supplementary Figure 6): δ 209.24, 160.45, 129.39, 112.02, 110.50, 105.90, 59.90, 45.87, 18.27, 14.37. Reaction conditions, including reaction temperature, reaction time, and solvents, are presented in Table 4.

**TABLE 4:** The conditions of synthesis of 1-(2, 6-dihydroxyphenyl) butane-1-one.

Entry	Substrate	Temp (°C)	Catalyst	Equiv of BBr_3_	Solvent	Yield (%)
1	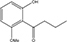	RT		1	DCM	30
2	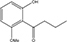	0		1	DCM	26
3	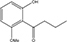	−20		1	DCM	20
4	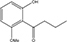	RT		2	DCM	35
5	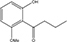	0		2	DCM	28
6	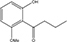	−20		2	DCM	25
7	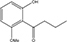	RT		3	DCM	51
8	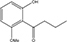	0		3	DCM	45
9	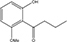	−20		3	DCM	40

## 3. Results

### 3.1. The inhibitory activity on pathogenic strains of compound 5

The crude ethyl acetate extract of *D. childiae* showed strong activity on the tested pathogenic strains. The 19 secondary metabolites isolated from endolichenic fungus were further investigated for antimicrobial activity. However, in this study, only compound 5, namely, 1-(2,6-dihydroxyphenyl) butane-1-one exhibited the antibacterial activity. Moreover, the compound showed the broad-spectrum antimicrobial activity.

**FIGURE 1:**
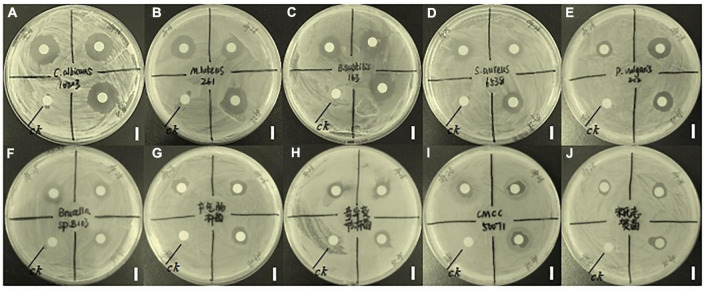
Inhibitory effects of compound 5 on 10 tested strains. The CK represents a blank control and the bar scale is 9 mm. Panel **(A)** represents the inhibitory effect of compound 5 on *C. albicans* 10213. Panel **(B)** represents the inhibitory effect of compound 5 on *M. luteus* 261. Panel **(C)** represents the inhibitory effects of compound 5 on *B. subtilis* 163. Panel **(D)** represents the inhibitory effect of compound 5 on *S. aureus* 6538. Panel **(E)** represents the inhibitory effect of compound 5 on *P. vulgaris* Z12. Panel **(F)** represents the inhibitory effect of compound 5 on *Brucella* sp. B103. Panel **(G)** represents the inhibitory effect of compound 5 on *E. aerogenes*. Panel **(H)** represents the inhibitory effect of compound 5 on *P. mirabilis*. Panel **(I)** represents the inhibitory effect of compound 5 on *S. typhimurium* CMCC 50071. Panel **(J)** represents the inhibitory effect of compound 5 on *S. sonnet*.

Compound 5 demonstrated inhibitory effects on 10 out of the 15 tested strains including Gram-positive bacteria, Gram-negative bacteria, and fungus. As shown in Figure 1, compound 5 shows strong inhibition activity against fungus *C. albicans* 10213 with an inhibition zone of more than 16.5 mm (Figure 1A). The inhibitory effects are moderate on *S. aureus* 6538, *M. luteus* 261, *B. subtilis* 163, and *P. vulgaris* Z12 with the inhibition zone of 11, 13, 13, and 13 mm, respectively, (Figures 1B–E) but the weak effect on *S. sonnet*, *Brucella* sp. B103, *E. aerogenes*, *P. mirabilis*, and *S. typhimurium* CMCC 50071 with an inhibition zone of 7.0–10.5 mm (Figures 1F–J).

### 3.2. MIC and MBC of compound 5 *in vitro* conditions

**FIGURE 2:**
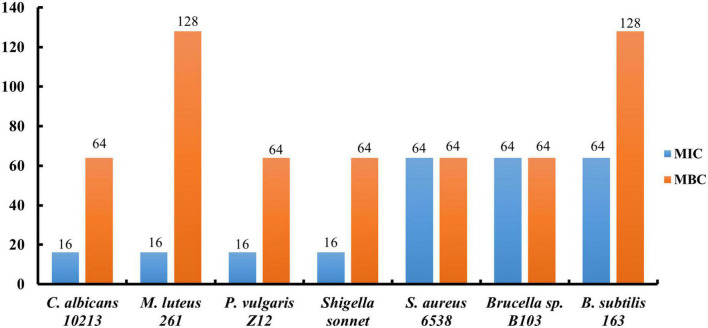
The MICs and MBCs of compound 5 for tested strains.

The minimum inhibitory concentration (MIC) and minimum bactericidal concentration (MBC) *in vitro* conditions were further measured to evaluate the antibacterial activities of compound 5. It was found that MIC of *C. albicans* 10213, *M. luteus* 261, *P. vulgaris* Z12, *S. sonnet*, *S. aureus* 6538, *Brucella* sp. B103, *B. subtilis* 163 were 16, 16, 16, 16, 64, 64, and 64 μg/ml, respectively. Furthermore, MBC of *C. albicans* 10213, *M. luteus* 261, *P. vulgaris* Z12, *S. sonnet*, *S. aureus* 6538, *Brucella* sp. B103, *B. subtilis* 163 are 64, 128, 64, 64, 64, 64, 128 μg/ml, respectively, as shown in Figure 2.

### 3.3. The growth curve of three strain

**FIGURE 3:**
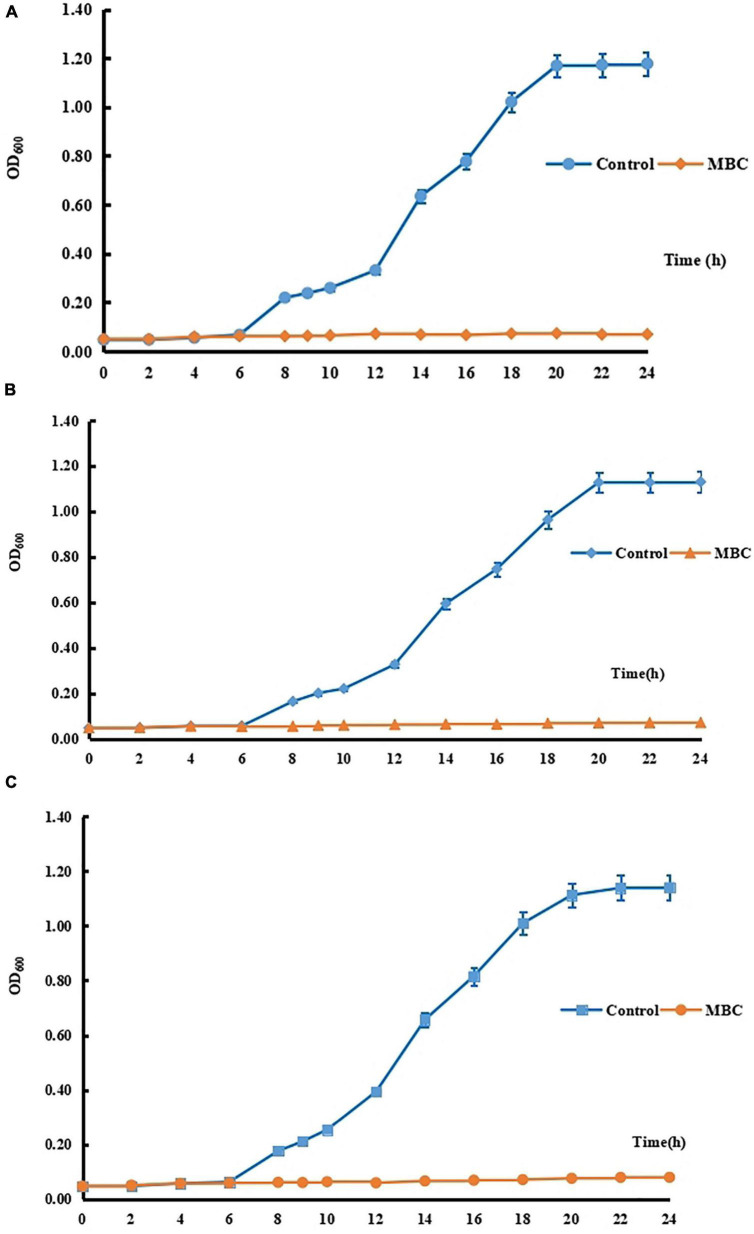
**(A)** The growth curve of *C. albicans* 10213. **(B)** The growth curve of *P. vulgaris* Z12. **(C)** The growth curve of *S. aureus* 6538.

In this study, to study the antimicrobial mechanism of compound 5 in this experiment, three strains of Gram-positive bacteria *S. aureus* 6538, Gram-negative bacteria *P. vul*garis Z12, and fungus *C. albicans* 10213 were selected. The growth curves of *S. aureus* 6538, *P. vulgaris* Z12, and *C. albicans* 10213 have been determined as shown in Figures 3A–C. It was found that under normal conditions, the three tested strains entered the logarithmic growth period after 10 h of culture, and the OD600 increased exponentially and became stable after 20 h. However, after the addition of compound 5 with MBC concentration, the OD600 did not change significantly and tended to increase by “0,” indicating that compound 5 could obviously inhibit the growth of the three pathogenic bacteria.

### 3.4. The content of nucleic acid

**FIGURE 4:**
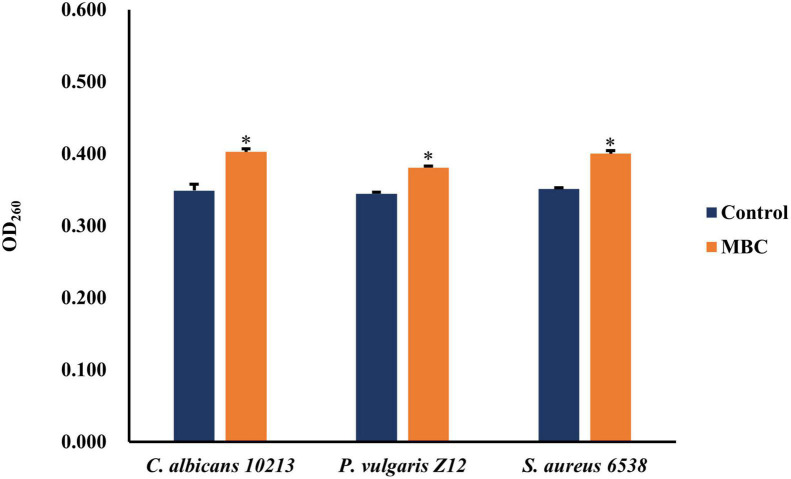
The effect of compound 5 on leakage of nucleic acid from tested strains. *Indicates a significant difference compared to the control group.

When the cell wall or cell membrane of microbes is damaged, the nucleic acid will permeate to the outside of the cell, leading to a higher content of nucleic acid. The effects of compound 5 with a concentration equal to the MBC on the permeability of the cell wall and cell membrane of *S. aureus* 6538, *P. vulgaris* Z12, and *C. albicans* 10213 were explored. Compared to the control group, the contents of nucleic acid in the test groups adding compound 5 are higher, as shown in Figure 4 and Table 5, which was statistically significant (*p* < 0.05). Compound 5 was effective at inhibiting the growth of all three strains, with a concentration of MBC. The results suggested that the ability of compound 5 to inhibit the growth of tested strains may be due to its ability to cause damage to the cell membrane and cell wall. This damage led to an increase in the permeability of the three tested strains, which in turn caused more nucleic acid to leak out of the cells.

**TABLE 5:** The effect of compound 5 on OD_260_ from tested strains.

	*C. albicans* 10213		*P. vulgaris* Z12		*S. aureus* 6538	
	**Control**	**MBC**	**Control**	**MBC**	**Control**	**MBC**
OD_260_ (*n* = 3)	0.349 ± 0.009	0.402 ± 0.004*	0.344 ± 0.002	0.381 ± 0.002*	0.351 ± 0.001	0.400 ± 0.004*

*Indicates a significant difference compared to the control group.

### 3.5. Determination of the content of protein

#### 3.5.1. The standard curve of bovine albumin (BSA)

**FIGURE 5:**
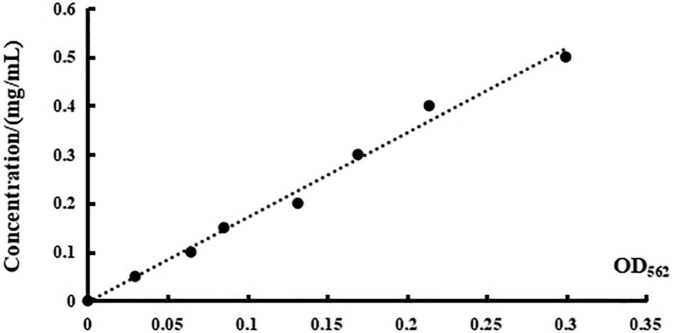
The standard curve of BSA.

A series of concentrations of BSA ranging from 0.05 mg/ml to 0.50 mg/ml was used as the ordinate, and the standard curve of standard BSA was formed with the corresponding OD_562_ value as the abscissa. There is a linear correlation between the concentration and the absorbance as depicted in Figure 5, as evidenced by the linear regression equation of *y* = 1.734*x*—0.003, with *R*^2^ = 0.990.

**FIGURE 6:**
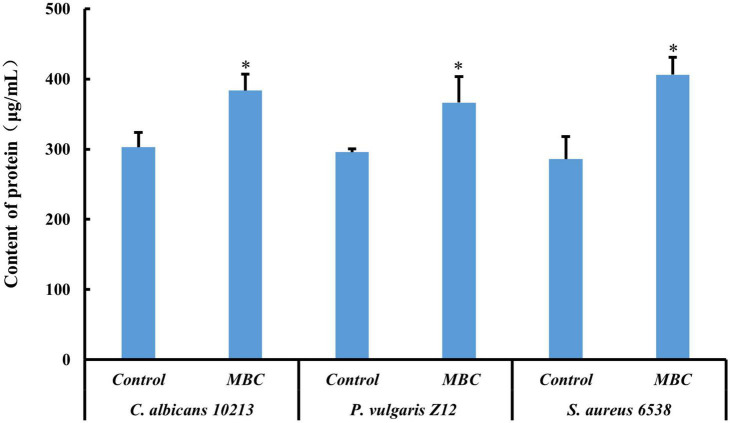
Effect of compound 5 on leakage of protein from the tested strains. *Indicates a significant difference compared to the control group.

The content of protein in pathogenic strains was determined by the BCA method. In alkaline conditions, the Cu^2+^ will be reduced to Cu^+^ by protein. The Cu^+^ and BCA reagent formed a purple complex, with two molecular BCA chelating one Cu^+^. The content of protein measured could be calculated by the standard curve of BSA. After adding compound 5 of MBC concentration, the OD value of *S. aureus* 6538, *P. vulgaris* Z12, and *C. albicans* 10213 increases as shown in Figure 6 and Table 6. The OD value of the three test strains at 562 nm was higher than that of the control group (*p* < 0.05), which was statistically significant. Compared to the control group, the content of protein in the test groups increased, indicating that the permeability of the cell membrane and cell wall of the three strains may have been damaged by compound 5, leading to protein leakage and increased content. These results were consistent with the influence on the nucleic acid.

**TABLE 6:** The effect of compound 5 on OD_562_ from tested strains.

	*C. albicans* 10213		*P. vulgaris* Z12		*S. aureus* 6538	
	**Control**	**MBC**	**Control**	**MBC**	**Control**	**MBC**
OD_562_ (*n* = 3)	0.176 ± 0.014	0.223 ± 0.015*	0.172 ± 0.002	0.213 ± 0.023*	0.167 ± 0.020	0.236 ± 0.016*

*Indicates a significant difference compared to the control group.

### 3.6. Determination of content of AKP

**FIGURE 7:**
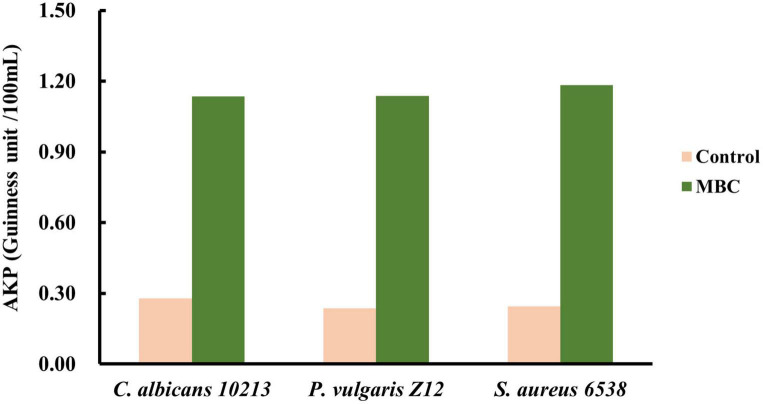
Effect of compound 5 on AKP activity from the tested strains.

Alkaline phosphatase is a glycoprotein containing zinc. In an alkaline environment, AKP catalyzes the conversion of disodium diphenyl phosphate to free phenols, which is then oxidized with 4-aminoantipyrine by potassium ferricyanide to form red quinone derivatives with a characteristic light absorption at 520 nm. As shown in Table 7, after adding compound 5 with MBC concentration, the OD value of the three kinds of tested strains, Gram-positive bacteria *S. aureus* 6538, Gram-negative bacteria *P. vulgaris* Z12 and fungus *C. albicans* 10213 at 520 nm is found to be higher than that of the control group (*p* < 0.05), which is statistically significant. Combined with the calculation formula, as shown in Figure 7, the AKP activity of the MBC group is significantly stronger than that of the control group, indicating that under MBC concentration, compound 5 may have damaged the cell walls and cell membranes of the three tested strains, leading to the dissolution of AKP and the enhancement of its activity.

**TABLE 7:** The effect of compound 5 on OD_520_ from tested strains.

	*C. albicans* 10213		*P. vulgaris* Z12		*S. aureus* 6538		Stander of phenol	Blank
	**Control**	**MBC**	**Control**	**MBC**	**Control**	**MBC**		
OD_520_ (*n* = 3)	0.090 ± 0.001	0.192 ± 0.002*	0.085 ± 0.002	0.192 ± 0.004*	0.086 ± 0.003	0.197 ± 0.002*	0.294 ± 0.003	0.057 ± 0.002

*Indicates a significant difference compared to the control group.

### 3.7. Synthesis of 2-hydroxy-6-methoxybenzaldehyde

The substrate 2,6-dihydroxybenzaldehyde and K_2_CO_3_ reacted for 3 and 6 h, respectively, at room temperature, 68 and 80°C in THF solvent. The highest yield of 2-hydroxy, 6-methoxybenzaldehyde was 78% at room temperature for 6 h.

Treatment of 2,6-dihydroxybenzaldehyde with CH_3_I in the presence of K_2_CO_3_ at room temperature in THF provided the desired product 2-hydroxy-6-methoxybenzaldehyde in 78% yield.

### 3.8. Synthesis of 1-(2-hydroxy -6-methoxyphenyl) butanol

The nucleophilic addition reaction of 2-hydroxy- 6-methoxybenzaldehyde with Grignard reagent propylmagnesium bromide produced the desired product 1-(2-hydroxy-6-methoxyphenyl) butanol in 70% yield under the optimal reaction conditions (compound 3).

### 3.9. Synthesis of 1-(2-hydroxy-6-methoxyphenyl) butanone

The variation of reaction conditions for secondary alcohol oxidation was carried out using different oxidants, such as PCC, IBX (2-iodoxybenzoic acid), and DMP (1,1,1-triacetoxy-1,1-dihydro-1,2-benziodoxol-3 (1*H*)-One). Among these screened oxidants, PPC was found to be the best for the reaction.

### 3.10. Synthesis of 1-(2,6-dihydroxyphenyl) butane-1-one

The methoxy group at C6 on the benzene ring of compound 4 was catalyzed into the hydroxyl group by oxidant BBr_3_ through nucleophilic substitution reaction to generate the target compound 5. At room temperature, the yield was higher than −20 and 0°C which reached 51%.

Deprotection of the methyl with BBr_3_ in anhydrous CH_2_Cl_2_ at room temperature afforded the desired product 5 in a yield of 51%.

## 4. Discussion

In recent years, due to the irrational use of antibiotics, the phenomenon of antibiotic resistance in various bacteria is consistently increasing and has become increasingly serious to human health, so it is important to explore new antibacterial active ingredients. Mycobionts not only coexist with photobionts but also with a large number of endophytes, which are considered as the hidden microbial resources. Due to the special habitat, endolichenic fungi could produce many secondary metabolites with novel structures or with significant activities, which is of great significance to provide a basis for exploring potential active compounds. Nineteen compounds were isolated from an endophytic fungus with antimicrobial activity, and only compound 5 showed strong and broad-spectrum antimicrobial activity.

According to the literature (Shen et al., 2015; Luo et al., 2019), if the cell membrane and cell wall of the test bacteria are damaged, it will lead to the leakage of nucleic acid, protein, and other substances, which will inhibit the growth of the test bacteria, which cause even death. In our study, compound 5 at MBC concentration could significantly inhibit the growth of *S. aureus* 6538, *P. vulgaris* Z12, and fungus *C. albicans* 10213. The increased dissolution of nucleic acid and protein, along with the enhancement of AKP activity, indicated that compound 5 inhibited the pathogenic microbes by affecting the permeability of the cell membrane and cell wall. These findings suggest that compound 5 may have the potential as an antimicrobial agent that can target a broad range of microorganisms.

Many natural products are highly favored due to their good biological activity, but the low content limits their further application. The chemical synthesis of natural products provided an alternative method. In this study, the target compound has been successfully synthesized through four steps of a chemical reaction. Oxidants PCC, 2-Iodoxybenzoic acid (IBX), and Dess Martin were screened in the synthesis of 1-(2-hydroxy, 6-methoxyphenyl) butanone (compound 4). PCC is a mild and selective oxidant. However, as the reaction proceeded, dark brown oily residues were generated that could wrap the products and led to a reduction in the yield of compound 4. IBX is another mild, highly selective, environmentally friendly, widely used, and selective oxidant but has a better solubility in dimethyl sulfoxide (DMSO). However, the boiling point of the solvent DMSO is higher while 1-(2, hydroxy, 6-methoxyphenyl) butanol is a reactant with a low boiling point. Moreover, the reaction needed to be carried out at room temperature which led to the low solubility of IBX in DMSO and low yield of compound 4 as the oxidant. Besides, Dess Martin could also be an efficient oxidant to oxidize 1-(2,hydroxy,6-methoxyphenyl) butanol into compound 3 at room temperature (Dess Martin, which is an oxidant, can oxidize primary alcohols to aldehydes). However, this oxidant is hydrophobic and needs to be added drop by drop at low temperatures with troublesome post-treatment that led to poor products. Herein, PCC was selected in the present study. In the final synthesis of the target product, BBr3 is used for demethylation reaction. One of the biggest disadvantages of BBr3 is that it is overly sensitive to air. Moreover, a large number of aerosols with pungent smells will affect the rate of aerosol deposition, leading to an incomplete reaction. Additionally, the pH of the extraction also had an impact on the yield of the products.

## 5. Conclusion

Compound 5, a secondary metabolite with broad-spectrum antimicrobial activity isolated from the endolichenic fungus from Guizhou province, showed inhibitory effects on 10 of the 15 tested strains, including Gram-positive bacteria, Gram-negative bacteria, and fungus. The MICs of compound 5 for *C. albicans* 10213, *M. luteus* 261, *P. vulgaris* Z12, *S. sonnet*, and *S. aureus* 6538 was 16 μg/ml, while the MBCs of *C. albicans* 10213, *P. vulgaris* Z12, *S. aureus* 6538, and *Brucella* sp. B103 were 64 μg/ml. Compound 5 could obviously inhibit the growth of *S. aureus* 6538, *Proteus* sp., and *C. albicans* 10213 at the concentration of MBC, likely by affecting the permeability of the cell wall and membrane. The results enriched the library of active strains and metabolites resources of endolichenic fungi. Moreover, the exploration of the chemical synthesis of active compounds was also achieved by four steps for the first time, providing new alternative methods for the excavation of antimicrobial agents.

## Data availability statement

The datasets presented in this study can be found in online repositories. The names of the repository/repositories and accession number(s) can be found in the article/Supplementary material.

## Author contributions

S-BF designed the experiment, supervised the study, and corrected the manuscript. XZ and M-YW performed the experiments. XZ, Q-FM, Q-PC, ZY, and S-BF analyzed the data. Q-FM and S-BF wrote the manuscript. All authors contributed to the article and approved the submitted version.
