# Supplementary material for: Chemical synthesis and mechanism of a natural product from endolichenic fungus with a broad-spectrum anti microorganism activity
Source: Front Microbiol. 2023 May 5;14:1168386. doi: 10.3389/fmicb.2023.1168386 (PMC10196465; doi:10.3389/fmicb.2023.1168386)
Supplement: Supplementary file 1 [file Data_Sheet_1.docx]

Supplementary Material

Chemical Synthesis and Mechanism of a Natural Product from Endolichenic Fungus with a Broad Spectrum Anti-microorganism Activity

Xuan Zhou ^1†^, Ming-Yi Wang ^1†^, Qian-Ping Cao ^1†^, Ze Yang ^1^, Qing-Feng Meng ^2, 3*^ and Shao-Bin Fu ^1*^

^1^ School of Pharmacy, Zunyi Medical University, Zunyi 563000, People’s Republic of China

^2^ School of Public Health, Zunyi Medical University, Zunyi 563000, People’s Republic of China

^3^ Centre of Excellence in Fungal Research, MeaFahLuangUnversity, Chiang Rai 57100, Thailand

*** Correspondence:**Corresponding Author
sbfu@zmu.edu.cn (Shao-Bin Fu)

Qfmeng@126.com

# Supplementary Data

# Supplementary Figures and Tables

# The ^1^H-NMR spectrum of 2


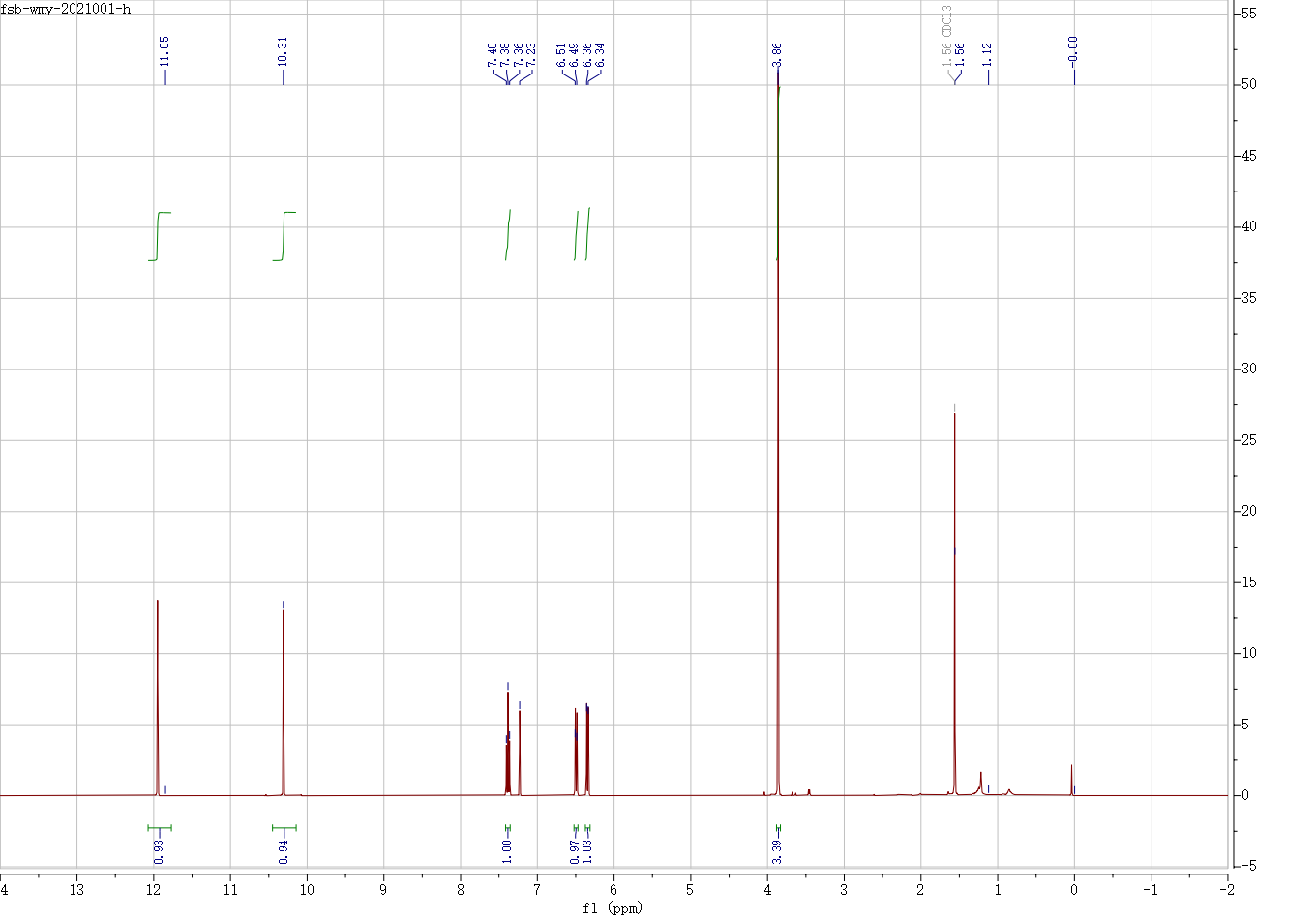


**Supplementary Figure 1.** ^1^H-NMR spectrum (400 MHz) of **2** measured in CDCl_3_ with a NMR instrument.

## The ^1^H-NMR spectrum of 3


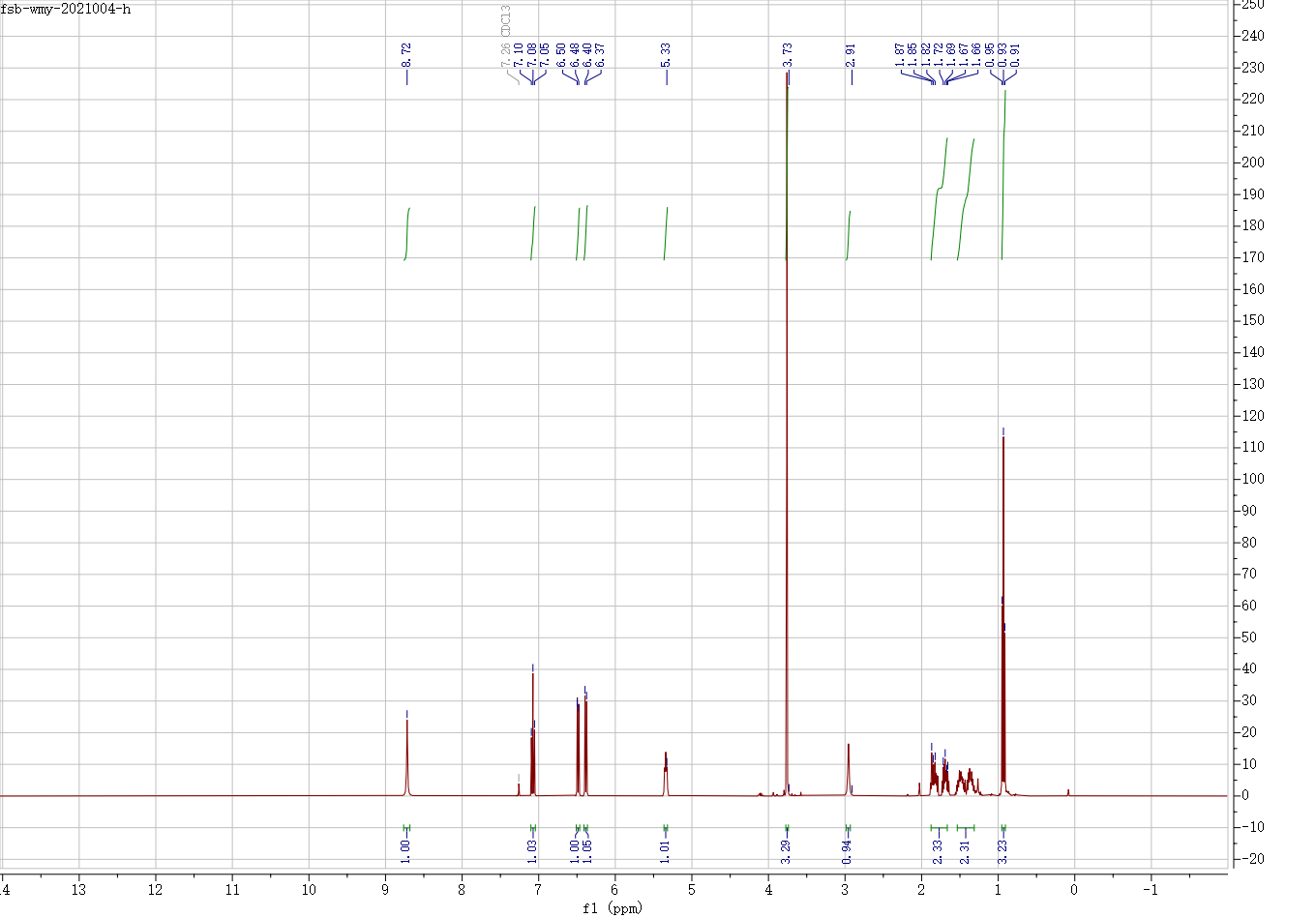


**Supplementary Figure 2.** ^1^H-NMR spectrum (400 MHz) of **3** measured in CDCl_3_ with a NMR instrument.

## The ^13^C-NMR spectrum of 3


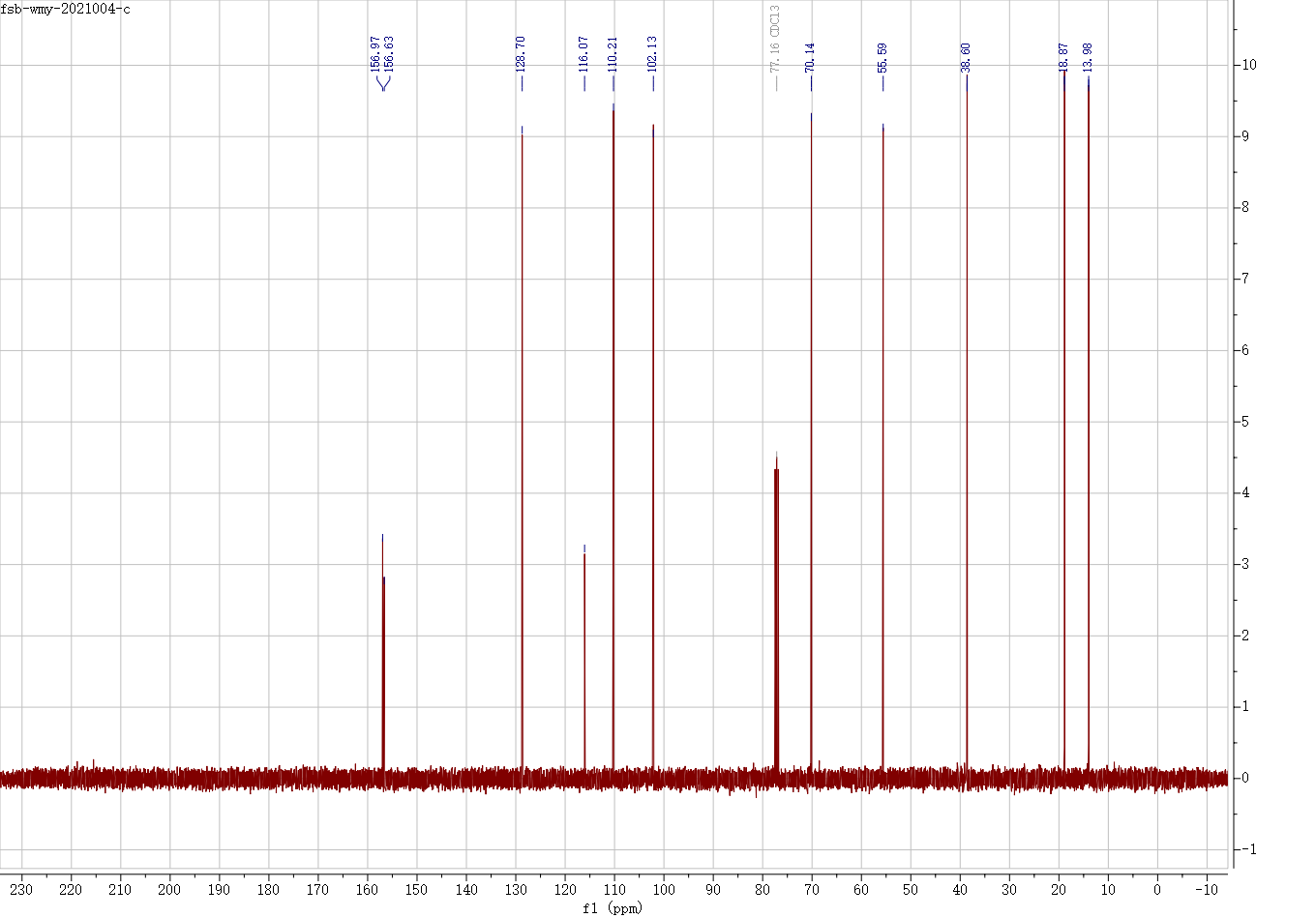


**Supplementary Figure 3.** ^13^C-NMR spectrum (100 MHz) of **3** measured in CDCl_3_ with a NMR instrument.

## The ^1^H-NMR spectrum of 4


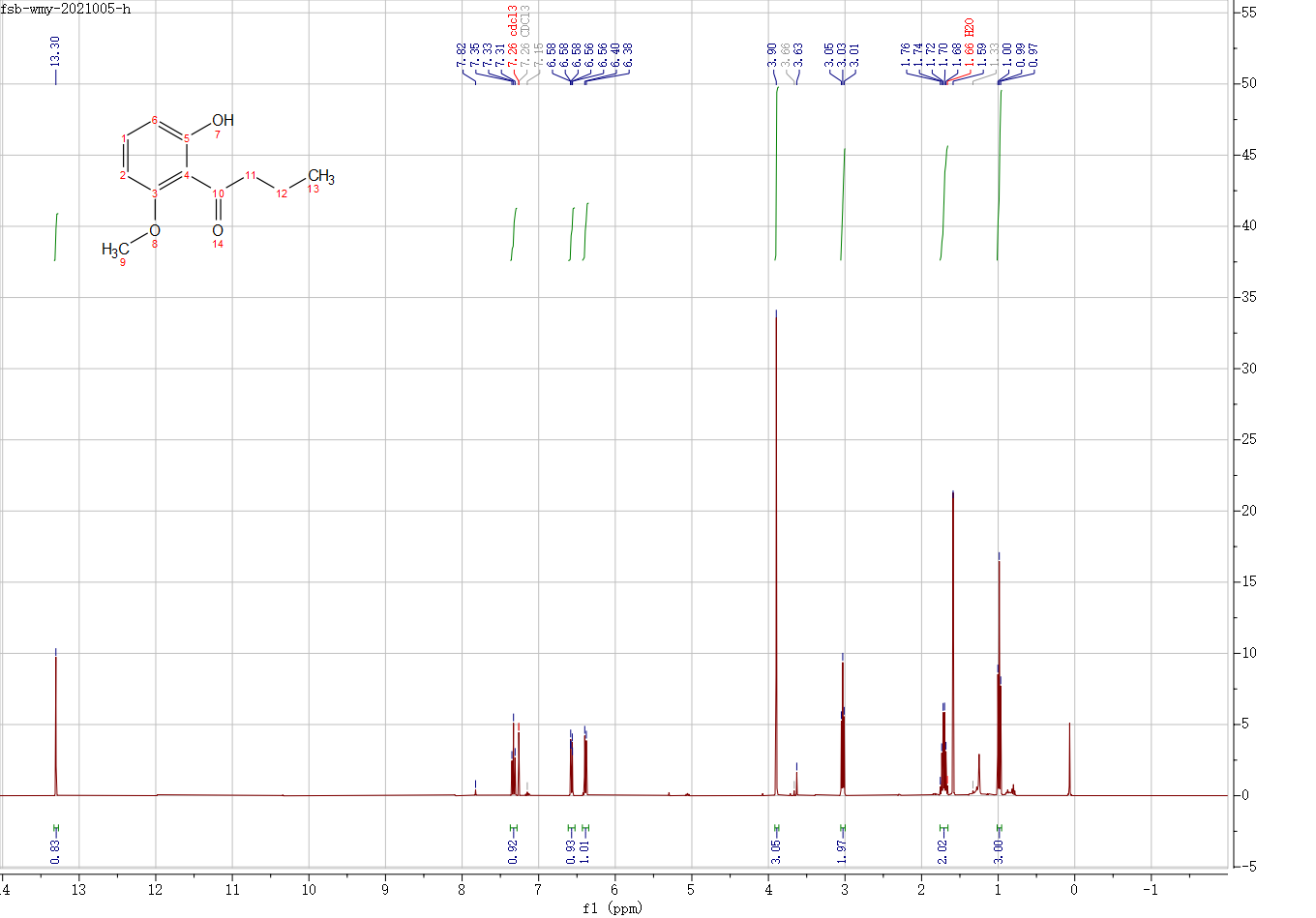


**Supplementary Figure 4.** ^1^H-NMR spectrum (400 MHz) of **4** measured in CDCl_3_ with a NMR instrument.

## The ^1^H-NMR spectrum of 5


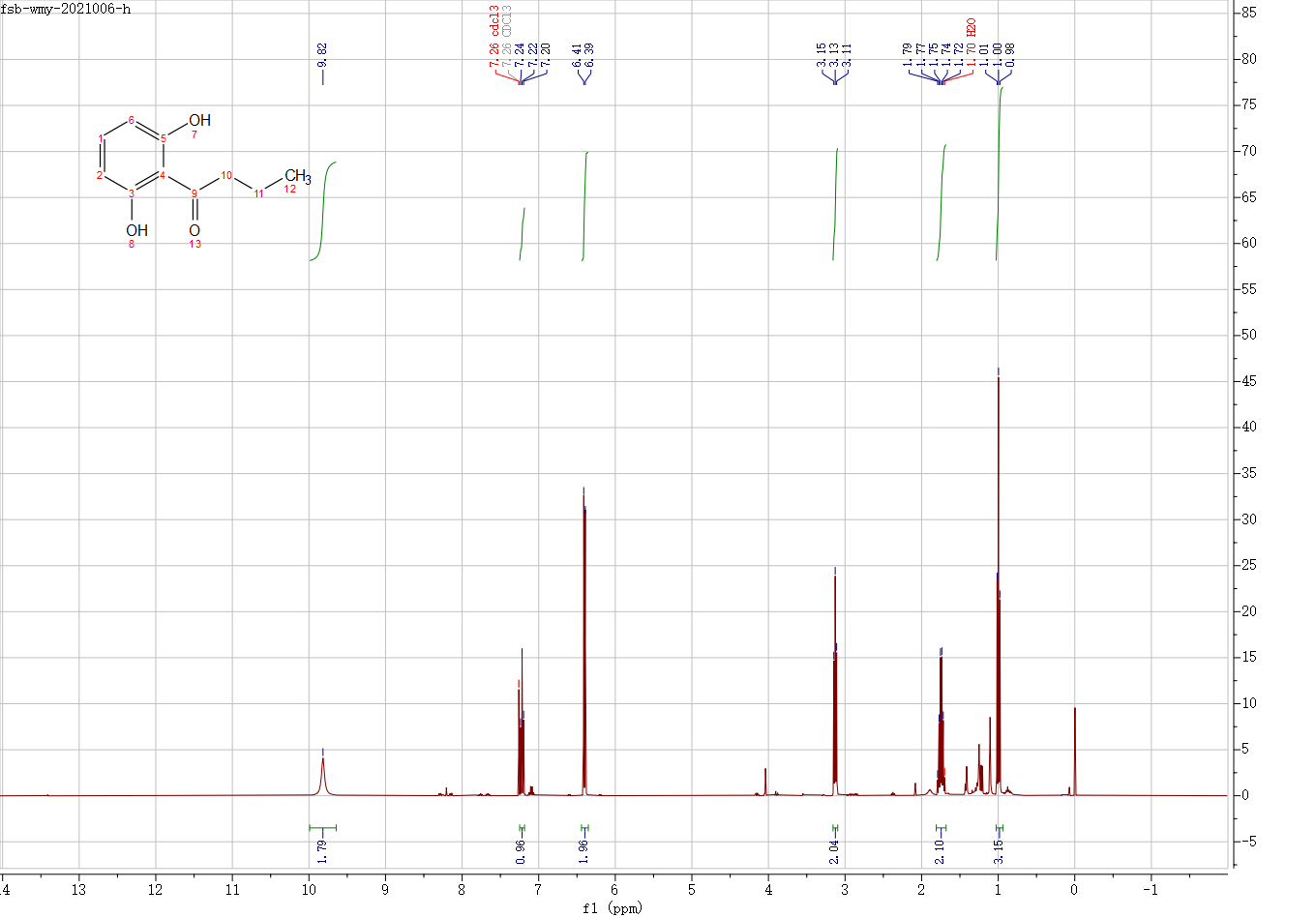


**Supplementary Figure 5.** ^1^H-NMR spectrum (400 MHz) of **5** measured in CDCl_3_ with a NMR instrument.

## The ^13^C-NMR spectrum of 5


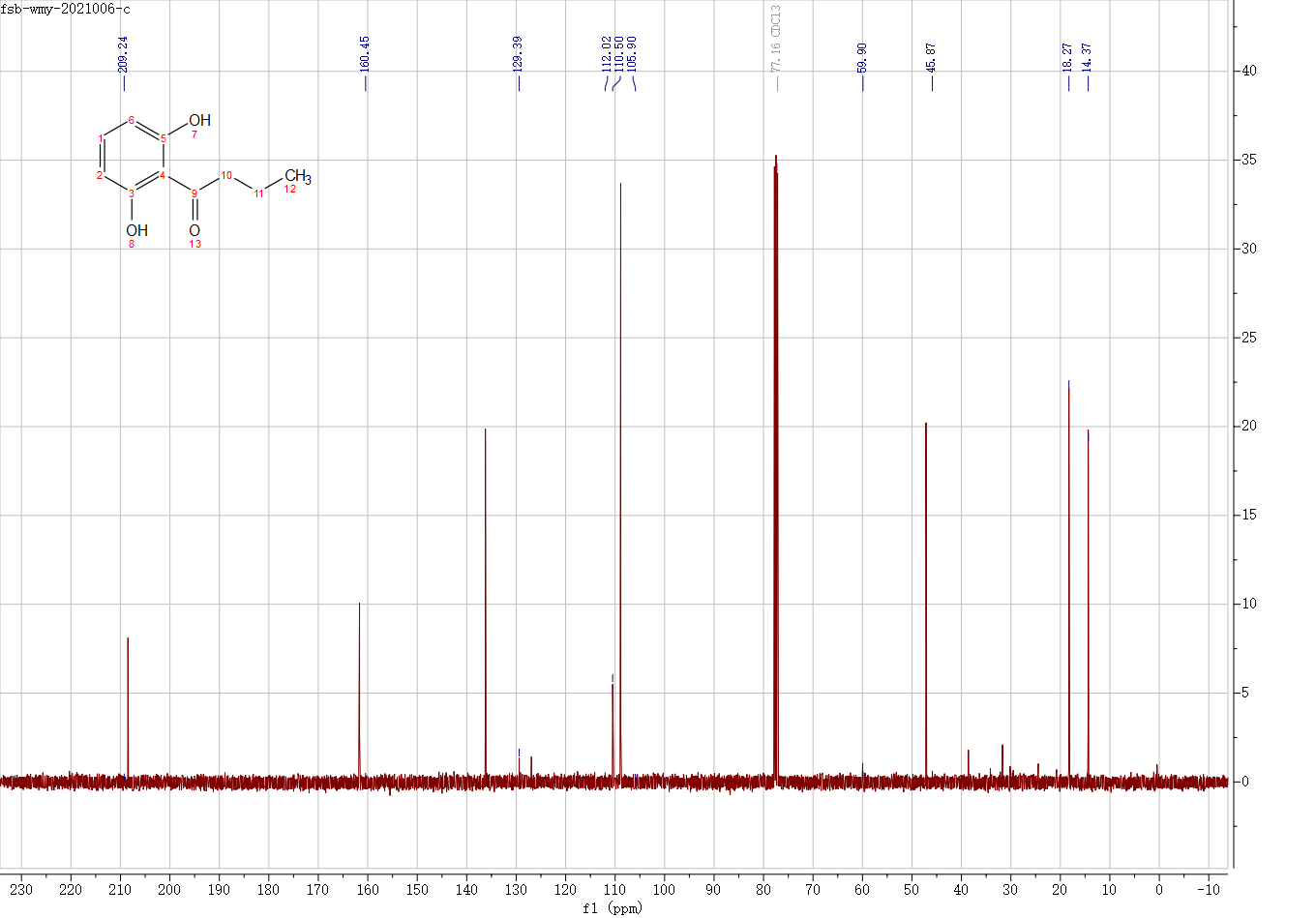


**Supplementary Figure 6.** ^13^C-NMR spectrum (100 MHz) of **5** measured in CDCl_3_ with a NMR instrument.

## Tables

Table 1 The reaction conditions for the synthesis of 2-hydroxy-6-methoxybenzaldehyde

| entry | Substrate | Temp (℃) | Time (h) | solvent | Yield (%) |
| --- | --- | --- | --- | --- | --- |
|  |  | RT | 3 | THF | 30 |
| 2 |  | 68 | 3 | THF | 20 |
| 3 |  | RT | 6 | THF | 78 |
| 4 |  | 68 | 6 | THF | 60 |
| 5 |  | RT | 6 | DMF | 25 |
| 6 |  | 80 | 6 | DMF | 28 |
| 7 |  | RT | 6 | DCM | 10 |

Table 2 The conditions of synthesis of 1 - (2-hydroxy-6-methoxyphenyl)butanol

| entry | substrate | Temp (℃) | Time (h) | solvent | Yield (%) |
| --- | --- | --- | --- | --- | --- |
| 1 |  | 0 | 3 | THF | 30 |
| 2 |  | -20 | 3 | THF | 25 |
| 3 |  | -78 | 3 | THF | 15 |
| 4 |  | 0 | 6 | y THF | 70 |
| 5 |  | -20 | 6 | THF | 55 |
| 6 |  | -78 | 6 | THF | 40 |

Table 3 The conditions of synthesis of -(2-hydroxy-6-methoxyphenyl) butanone

| entry | substrate | Time (h) | Oxidant | Yield (%) |
| --- | --- | --- | --- | --- |
| 1 |  | 2 | PCC | 30 |
| 2 |  | 4 | PCC | 27 |
| 3 |  | 6 | PCC | 25 |
| 4 |  | 2 | IBX | 15 |
| 5 |  | 4 | IBX | 18 |
| 6 |  | 6 | IBX | 20 |
| 7 |  | 2 | DMP | 17 |

Table 4 The conditions of synthesis of 1-(2,6-dihydroxyphenyl)butane-1-one

| entry | Substrate | temp(℃) | catalyst | equiv of BBr_3_ | solvent | yield(%) |
| --- | --- | --- | --- | --- | --- | --- |
| 1 |  | RT |  | 1 | DCM | 30 |
| 2 |  | 0 |  | 1 | DCM | 26 |
| 3 |  | -20 |  | 1 | DCM | 20 |
| 4 |  | RT |  | 2 | DCM | 35 |
| 5 |  | 0 |  | 2 | DCM | 28 |
| 6 |  | -20 |  | 2 | DCM | 25 |
| 7 |  | RT |  | 3 | DCM | 51 |
| 8 |  | 0 |  | 3 | DCM | 45 |
| 9 |  | -20 |  | 3 | DCM | 40 |

**Table 5.** The effect of compound **5** on OD_260_ from tested strains

|  | *C. albicans* 10213 | | | *P. vulgaris* Z12 | | | | | *S. aureus* 6538 | | |
| --- | --- | --- | --- | --- | --- | --- | --- | --- | --- | --- | --- |
|  | Control | MBC | Control | | MBC | | Control | | | MBC | |
| OD_260_  (n=3) | 0.349±0.009 | 0.402±0.004^*^ | 0.344±0.002 | | | 0.381±0.002^*^ | | 0.351±0.001 | | | 0.400±0.004^*^ |

**Table 6.** The effect of compound **5** on OD_562_ from tested strains

|  | *C. albicans* 10213 | | | *P. vulgaris* Z12 | | | | | *S. aureus* 6538 | | |
| --- | --- | --- | --- | --- | --- | --- | --- | --- | --- | --- | --- |
|  | Control | MBC | Control | | MBC | | Control | | | MBC | |
| OD_562_  (n=3) | 0.176±0.014 | 0.223±0.015^*^ | 0.172±0.002 | | | 0.213±0.023^*^ | | 0.167±0.020 | | | 0.236±0.016^*^ |

**Table 7.** The effect of compound **5** on OD_520_ from tested strains

|  | *C. albicans* 10213 | | | *P. vulgaris* Z12 | | | | | *S. aureus* 6538 | | | Stander of phenol | blank |  |
| --- | --- | --- | --- | --- | --- | --- | --- | --- | --- | --- | --- | --- | --- | --- |
|  | Control | | MBC | | | Control | | MBC | | Control | MBC |  |  |  |
| OD_520_  (n=3) | 0.090± 0.001 | 0.192± 0.002^*^ | | | 0.085±0.002 | | 0.192± 0.004^*^ | | | 0.086±0.003 | 0.197± .002^*^ | 0.294± 0.003 | 0.057±0.002 | |
